# Atrial Flutter as a Late Presentation of Scimitar Vein in an Adult With Prior ASD Repair

**DOI:** 10.1002/ccr3.72842

**Published:** 2026-06-02

**Authors:** Fereshte Rastegarnasab, Zahra Teimouri‐Jervekani, Keyvan Khojaste, Azam Soleimani

**Affiliations:** ^1^ Hypertension Research Center, Cardiovascular Research Institute Isfahan University of Medical Sciences Isfahan Iran; ^2^ Cardiac Rehabilitation Research Center, Cardiovascular Research Institute Isfahan University of Medical Sciences Isfahan Iran; ^3^ Student Research Committee Isfahan University of Medical Sciences Isfahan Iran; ^4^ Chamran Cardiovascular Medical and Research Hospital Isfahan University of Medical Sciences Isfahan Iran

**Keywords:** adult congenital heart disease, anomalous pulmonary vein, atrial flutter, scimitar vein

## Abstract

Adult patients with unexplained atrial arrhythmias and right heart dilation should prompt consideration of congenital anomalies such as scimitar vein, even decades after atrial septal defect repair. Early recognition using multi‐modality imaging may prevent delayed diagnosis and progressive cardiopulmonary complications.

## 1 Introduction

The scimitar vein represents a rare congenital cardiovascular anomaly classified as a form of partial anomalous pulmonary venous return (PAPVR). It is characterized by anomalous drainage of one or more right pulmonary veins into the inferior vena cava (IVC), either above or below the diaphragm, resulting in a left‐to‐right shunt. The estimated incidence is approximately 1–3 per 100,000 live births, with a slight female predominance [1, 2]. This condition is frequently associated with an atrial septal defect (ASD) [3].

Clinical presentation varies widely depending on disease severity and the presence of associated anomalies [4]. It ranges from severe symptoms in infancy, often requiring urgent intervention to augment pulmonary venous return, to milder or even asymptomatic forms in adulthood that are frequently detected incidentally on imaging [2].

In infancy, symptoms may include tachypnea, recurrent pneumonia, failure to thrive, and signs of heart failure. In contrast, adults may present with palpitations, chest pain, or dyspnea [5].

Herein, we report a 52‐year‐old woman with a history of surgical ASD closure at the age of 17, in whom a scimitar vein was incidentally diagnosed during evaluation for atrial flutter.

## 2 Case History/Examination

A 52‐year‐old woman presented with a complaint of sudden‐onset paroxysmal palpitations at rest, which resolved within 24 h.

She had a history of surgical closure of an ASD at age 17, although no surgical records were available. Additionally, her medical history included controlled hypertension and total thyroidectomy performed three years prior due to papillary thyroid carcinoma.

## 3 Differential Diagnosis, Investigations and Treatment

The initial electrocardiogram revealed regular narrow QRS tachycardia (heart rate = 150 BPM) along with incomplete right bundle branch block (RBBB) (Figure 1A). A diagnosis of atrial flutter with a 2:1 ventricular response was confirmed following adenosine administration. Subsequently, sinus rhythm was restored in the electrophysiology clinic, where the patient was evaluated for potential further management (Figure 1B).

**FIGURE 1:**
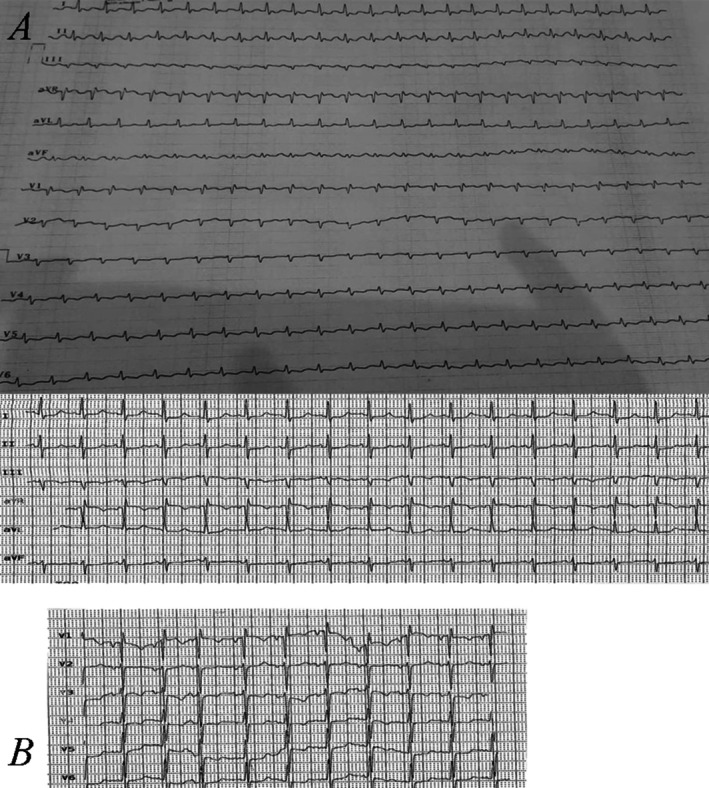
(A) A regular narrow QRS tachycardia associated with an incomplete right bundle branch block. Atrial Flutter with a 2:1 ventricular response was confirmed after adenosine injection. (B) Normal sinus rhythm.

Echocardiographic findings revealed a normal left ventricular (LV) size and function, with an LV ejection fraction of 55% (measured by the biplane Simpson method). Moderate right ventricular (RV) enlargement was noted, with preserved RV systolic function, as well as mild bi‐atrial enlargement. There was up to moderate mitral and tricuspid regurgitation, a tricuspid regurgitation gradient of 40 mmHg, a dilated main pulmonary artery, and a pulmonary artery pressure (PAP) of 45 mmHg. A venous agitated saline study showed no bubble passage through the repaired interatrial septum (IAS) at rest and during the Valsalva maneuver. The QP/QS ratio was calculated to be 1.5 (Video 1).

**VIDEO 1:** Echocardiogram demonstrating normal left ventricle (LV) size with normal LV function, moderate right ventricle (RV) enlargement, preserved RV systolic function, and mild bi‐atrial enlargement. Video content can be viewed at https://onlinelibrary.wiley.com/doi/10.1002/ccr3.72842.

Chest X‐ray revealed a tubular structure paralleling the right border of the heart, resembling a Turkish sword (scimitar) (Figure 2A).

**FIGURE 2:**
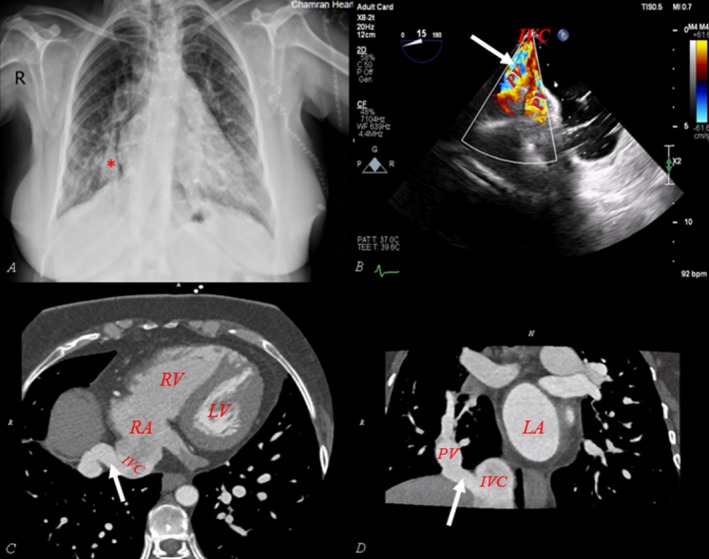
(A) Chest X‐ray demonstrating a tubular structure paralleling the right border of the heart in the shape of a Turkish sword (the scimitar vein pointed with a star), (B) Lower esophageal trans‐esophageal echocardiography view demonstrating right pulmonary veins (PV) drainage to the inferior vena cava (IVC). The arrow shows the right PV entering the IVC. (C) Axial, and (D) Frontal views of computed tomography angiography demonstrating the drainage of all right PVs to the IVC and mild non‐obstructive coronary artery disease. The arrow shows the scimitar vein entering the IVC. IVC, inferior vena cava; LA, left atrium; LV, left ventricle; PV, pulmonary vein; RA, right atrium; RV, right ventricle.

Transesophageal echocardiography (TEE) demonstrated normal drainage of the left PVs into the left atrium (LA). However, the right PVs were found to drain into the IVC via the scimitar vein (Figure 2B) (Videos 2 and 3).

**VIDEO 2:** Trans‐esophageal echocardiogram demonstrating normal drainage of left pulmonary veins to left atrium (LA). Video content can be viewed at https://onlinelibrary.wiley.com/doi/10.1002/ccr3.72842.

**VIDEO 3:** Trans‐esophageal echocardiogram demonstrating right pulmonary veins (PV) drainage to the inferior vena cava (IVC). Video content can be viewed at https://onlinelibrary.wiley.com/doi/10.1002/ccr3.72842.

Computed tomography angiography (CTA) further confirmed drainage of all right PVs into the IVC and also revealed mild non‐obstructive coronary artery disease (Figure 2C,D) (Videos 4, 5, 6). Surgical correction for partial anomalous pulmonary venous connection (PAPVC) was recommended.

**VIDEO 4:** Axial. Video content can be viewed at https://onlinelibrary.wiley.com/doi/10.1002/ccr3.72842.

**VIDEO 5:** Frontal. Video content can be viewed at https://onlinelibrary.wiley.com/doi/10.1002/ccr3.72842.

**VIDEO 6:** Sagittal views of computed tomography angiography demonstrating the drainage of all right PVs to the IVC and mild nonobstructive coronary artery disease. Video content can be viewed at https://onlinelibrary.wiley.com/doi/10.1002/ccr3.72842.

Pulmonary vascular resistance (PVR) was calculated with an echocardiography‐based formula published by Abbas et al. [6]. Regarding the calculated PVR < 2 and unavailable catheter appropriate for right heart catheterization (RHC) during the patient hospitalization, RHC was not performed.

Finally, in view of the patient's symptoms and the significant left‐to‐right shunt, surgical PAPVC correction was scheduled [7].

The right pulmonary venous return was rerouted to the LA by enlarging the ASD and using a pericardial patch to baffle the anomalous veins through the ASD, under cardiopulmonary bypass. The vertical vein to the IVC was closed. Both the right and left atria were enlarged.

## 4 Conclusion and Results (Outcome and Follow‐Up)

Postoperative echocardiographic evaluation showed a reduction in RV size, PAP, and the QP/QS ratio, along with successful redirection of the abnormal PVs into the LA with no residual shunt.

The patient was discharged in stable condition, with no arrhythmias or postoperative complications.

During 18 months follow‐up, no symptoms or documented arrhythmia were detected. The patient had no complaint of dyspnea or any other related symptoms.

Written informed consent was obtained from the patient for the publication of this case report and the associated personal information.

## 5 Discussion

Our case describes an adult woman presenting with tachyarrhythmia, who was incidentally diagnosed with a complex scimitar vein associated with pulmonary hypertension and subsequently underwent surgical correction of PAPVC.

The anomalous PV most commonly drains into the infradiaphragmatic IVC and less frequently into the suprahepatic segment of the IVC, hepatic veins, portal vein, azygous vein, coronary sinus, or the right atrium [8].

Previous adult reports have predominantly described asymptomatic patients incidentally diagnosed with PAPVR. In contrast, symptomatic cases typically present with progressive dyspnea, cough, or chest discomfort. Initial presentation with tachyarrhythmias is uncommon and has been reported only in a limited number of cases (Table 1) [3, 4, 9, 10, 11, 12, 13, 14, 15, 16, 17, 18, 19, 20, 21, 22].

**TABLE 1:** Reports of adult scimitar vein since 2020.

No.	Author	Year	Sex	Age	Presentation	Diagnosis	Management	Outcome
1	Tanabe et al. [8]	2026	M	47	Progressive dyspnea on exertion Additional stroke‐like symptoms	Hypoplastic right lung Infra‐diaphragmatic scimitar vein	Surgical repair	Improved well
2	Parkash et al. [9]	2026	F	21	Asymptomatic	Hypoplastic right lung Cardiac dextroposition PAPVR draining into the IVC (scimitar sign) Horseshoe lung configuration	Conservative	N/A
3	Nisa et al. [10]	2025	M	33	Initially, a left‐sided chest pain Later, asymptomatic	Horseshoe lung	Conservative	N/A
4	Kinjo et al. [11]	2025	M	52	Persistent AF	Scimitar syndrome Arrhythmia‐induced cardiomyopathy due to atrial fibrillation	Circumferential PV isolation No surgical repair	Improved well
5	Franca et al. [12]	2025	M	59	Severe dizziness and hypertensive emergency	Incomplete scimitar syndrome	Conservative	N/A
6	Dawadi et al. [4]	2024	F	40	Dry cough Exertional dyspnea	Scimitar syndrome	N/A	N/A
7	Shamal et al. [13]	2024	F	26	Recurrent pulmonary infection Fever Cough Wheezing	Scimitar syndrome	Conservative	Variable responses
8	Luknar et al. [14]	2024	F	50	Dyspnea on exertion	ASD Scimitar syndrome	Simultaneous ASD and scimitar vein surgical repair	Significant stenosis of the re‐implanted anomalous pulmonary vein finally improved well
9	Liu et al. [15]	2024	F	67	Cough and dyspnea	Scimitar syndrome with PAH	Conservative	Improved well
10	Samarawickrama et al. [3]	2023	F	67	Worsening shortness of breath Ankle edema Commencement of heart failure medication	Partial anomalous pulmonary venous drainage in the right upper pulmonary vein Large ASD Absent right lower pulmonary vein	Conservative (The patient declined surgical correction.)	Died due to complications of stroke following missing warfarin for a week
11	Ren et al. [16]	2023	F	36	Progressive left‐sided chest pain Repeated pulmonary infection Cough and expectoration	Horseshoe lung	N/A	N/A
12	Meliota et al. [17]	2022	M	21	Syncope	Double‐drainage scimitar‐like syndrome	Surgical repair	Improved well
13	Robledo et al. [18]	2022	F	27	Dyspnea Heart palpitations Lower extremity edema	Scimitar syndrome Arterial pulmonary hypertension	Conservative	Improved
14	Mounir et al. [19]	2020	M	22	New York Heart Association (NYHA) functional class 2 Transient palpitations	Scimitar syndrome	Surgical repair	Improved well
15	Mounir et al. [19]	2020	F	57	Chest discomfort Dyspnea on exertion	Scimitar syndrome Moderate right lung hypoplasia	Surgical repair	Improved well
16	Marlene et al. [20]	2020	M	69	Worsening acute over chronic progressive exertional dyspnea Palpitations (Recurrent AF)	Scimitar syndrome	Surgical repair	N/A
17	Palaniappan et al. [21]	2020	F	43	Progressive exertional dyspnea	Scimitar syndrome	Suggested heart catheterization, but not done due to financial issues	N/A

Right atrial volume overload resulting from the scimitar vein may lead to complications such as pulmonary hypertension and atrial tachyarrhythmias, including atrial fibrillation and atrial flutter. Also, prior studies have suggested that abnormal electrical activity originating from anomalous PVs may act as a trigger for premature atrial complexes [13, 23]. Recurrent pulmonary infections and congestive heart failure have also been reported [1, 2]. Therefore, timely diagnosis and appropriate management are essential to prevent these complications.

Severe pulmonary arterial hypertension results in RV pressure load, RA enlargement, and hypertrophy. This can provide the anatomic and electrophysiologic substrate for macro‐reentrant atrial flutter [24]. Therefore, RA enlargement and remodeling induced by the PAPVR, chronic left‐to‐right shunt, and pulmonary hypertension consistently associate as arrhythmogenic contributors.

The diagnostic evaluation of the scimitar vein typically begins with chest radiography [5]. However, advanced imaging modalities, including cardiac computed tomography, cardiac magnetic resonance imaging, and echocardiography, are preferred for detailed assessment of extracardiac vascular anatomy, confirmation of the diagnosis, and evaluation of the underlying cardiopulmonary status [2, 13, 23].

Management strategies depend on symptom severity and associated anomalies. Asymptomatic adults or older children with a single anomalous PV generally require conservative management with close follow‐up. In contrast, surgical and percutaneous interventions are the definitive treatment options for patients with a significant left‐to‐right shunt, symptomatic pulmonary hypertension, right heart failure, recurrent pulmonary infections, or those undergoing concomitant repair of other major cardiac lesions [2, 3, 7, 13].

However, the risk of postoperative complications, such as venous stenosis requiring reintervention, thrombosis, atrial fibrillation, and sinus node dysfunction, must be carefully considered [13]. Therefore, evaluation by a multidisciplinary team is often warranted.

In conclusion, this case underscores the importance of considering congenital anomalies such as partial anomalous pulmonary venous return (PAPVR) in adults presenting with unexplained cardiac symptoms. A high index of suspicion among clinicians and cardiologists is essential to ensure accurate diagnosis, guide appropriate management, and improve clinical outcomes.

## Author Contributions

**Fereshte Rastegarnasab:** investigation, writing – original draft, writing – review and editing. **Zahra Teimouri‐Jervekani:** conceptualization, project administration, supervision, writing – review and editing. **Keyvan Khojaste:** investigation, writing – original draft, writing – review and editing. **Azam Soleimani:** conceptualization, supervision, writing – review and editing.

## Funding

The authors have nothing to report.

## Ethics Statement

This manuscript has been ethically approved by the “Ethics Committee of Isfahan University of Medical Science, Isfahan, Iran”.

## Consent

Written informed consent was obtained from the patient for the publication of this case report and accompanying images.

## Conflicts of Interest

The authors declare no conflicts of interest.

## Data Availability

The data supporting this study are available upon request from the corresponding author.
